# Small but lethal: cystic tumour of the atrioventricular node—a case report

**DOI:** 10.1093/ehjcr/ytag686

**Published:** 2026-09-22

**Authors:** Jessica V Yao, Homa Forotan, Geoffrey Lee, William Wilson, Kai En Leong

**Affiliations:** Department of Cardiology, The Royal Melbourne Hospital, 300 Grattan Street, Parkville, 3050 Victoria, Australia; Department of Medicine, Dentistry and Health Sciences, The University of Melbourne, Grattan Street, Parkville, 3010 Victoria, Australia; Department of Cardiology, The Royal Melbourne Hospital, 300 Grattan Street, Parkville, 3050 Victoria, Australia; Department of Cardiology, The Royal Melbourne Hospital, 300 Grattan Street, Parkville, 3050 Victoria, Australia; Department of Cardiology, The Royal Melbourne Hospital, 300 Grattan Street, Parkville, 3050 Victoria, Australia; Department of Cardiology, The Royal Melbourne Hospital, 300 Grattan Street, Parkville, 3050 Victoria, Australia

**Keywords:** multimodal imaging, cystic tumour of the atrioventricular node, cardiac tumours, cardiac computer tomography, cardiac magnetic resonance imaging, case report

## Abstract

**Background:**

Cystic tumours of the AV node (CTAVN) are exceptionally uncommon congenital cardiac tuours, with an incidence of 0.0017-0.03%.

**Case summary:**

A 47-year-old woman with recurrent symptomatic tachycardia underwent an ablation. Coronary sinus was noted to be enlarged. Subsequent cardiac CT revelead a well-circumscribed cystic lesioni in the Triangle of Koch, suspicious of a CTAVN. Multimodal imaging, including echocardiography, coronary angiography and cardiac MRI confirmed a non-vascular, encapsulated lesion with imaging characteristics consistent with a benign cystic mass. Given the risk of sudden cardiac death, surgical resection was performed. Histopathology confirmed CTAVN. The postoperative course as complicated by complete heart block requiring pacemaker implantation, with no recurrence at one year.

**Discussion:**

CTAVN is a rare but clinically significant benign tumour associated with conduction abnormalities and malignant arrhythmias. This care highlights the importance of multimodal imaging in diagnosis and the role of early surgical intervention to mitigate life-threatening complications.

Learning points

- Cystic tumours of the atrioventricular node are histologically benign but confer high risk of sudden death due to complete heart block or ventricular arrhythmias.
- Cardiac CT and MRI play a crucial role in localizing and characterizing cystic tumours of the atrioventricular node.

## Introduction

Cystic tumour of the atrioventricular node (CTAVN), also known as mesothelioma of the AV node, is a rare congenital cardiac tumour. Despite being histologically benign, CTAVN is the most common primary cardiac tumour implicated in sudden cardiac death.^1^

## Summary figure

**Figure:**
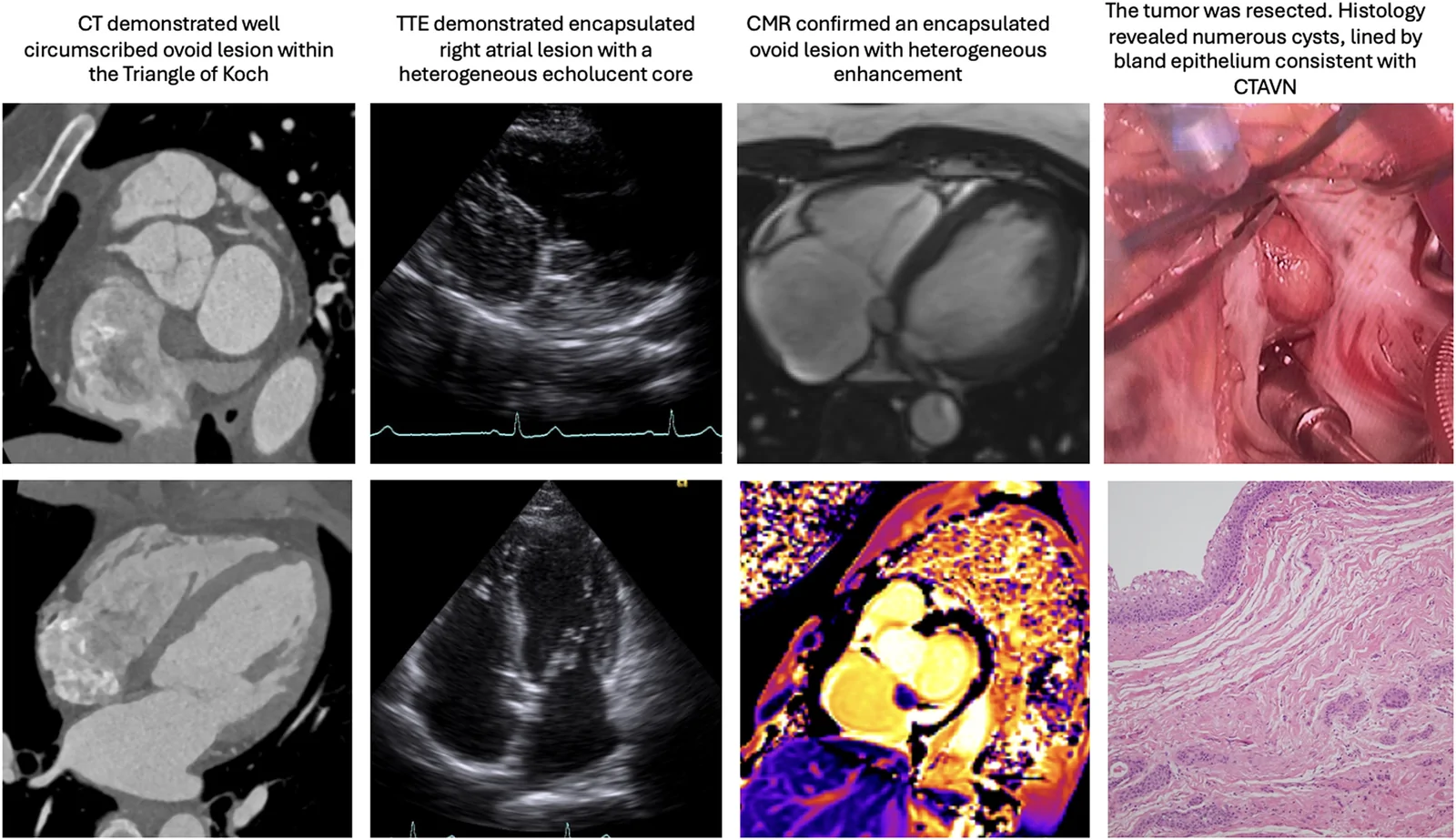


## History of presentation

A 47-year-old female presented with recurrent symptomatic supraventricular tachycardia (SVT), prompting multiple hospital admissions. There was no history of syncope. Past medical history included dyslipidaemia and gastro-oesophageal reflux disease. Medications included metoprolol, rosuvastatin, and esomeprazole. Baseline electrocardiogram demonstrated sinus rhythm, normal axis and a PR interval of 200 ms. She proceeded to SVT ablation under general anaesthesia. She had an easily inducible AV node re-entrant tachycardia (AVNRT). The slow pathway was successfully ablated. However, a further junctional rhythm was noted over a large coronary sinus (CS) ostium. After 10 minutes of ablation, the AVNRT was noted to be less inducible. Given close proximity to the AV node, no further ablation was attempted.

Cardiac computed tomography (CT) was arranged to assess the cardiac venous anatomy. This revealed an unexpected well circumscribed ovoid lesion in the right atrium within the Triangle of Koch, measuring 13 × 16 × 21 mm (mediolateral × craniocaudal × anteroposterior dimensions), suspicious for a cystic tumour of the atrioventricular node (CTAVN) (*Figure 1A–C*). Pre-contrast imaging (*Figure 1D*) showed no lesion calcification with overall low attenuation (mean 16 ± 14 HU). Borderline enhancement was seen on post-contrast imaging (mean 44 ± 28 HU vs. basal lateral LV 98 ± 31 HU and epicardial fat −58 ± 31 HU). The remainder of the study was unremarkable with normal cardiac venous anatomy, no pericardial effusion nor lymphadenopathy. There was no anomalous nor atheromatous coronary disease and no other significant extra-cardiac findings.

**Figures 1:**
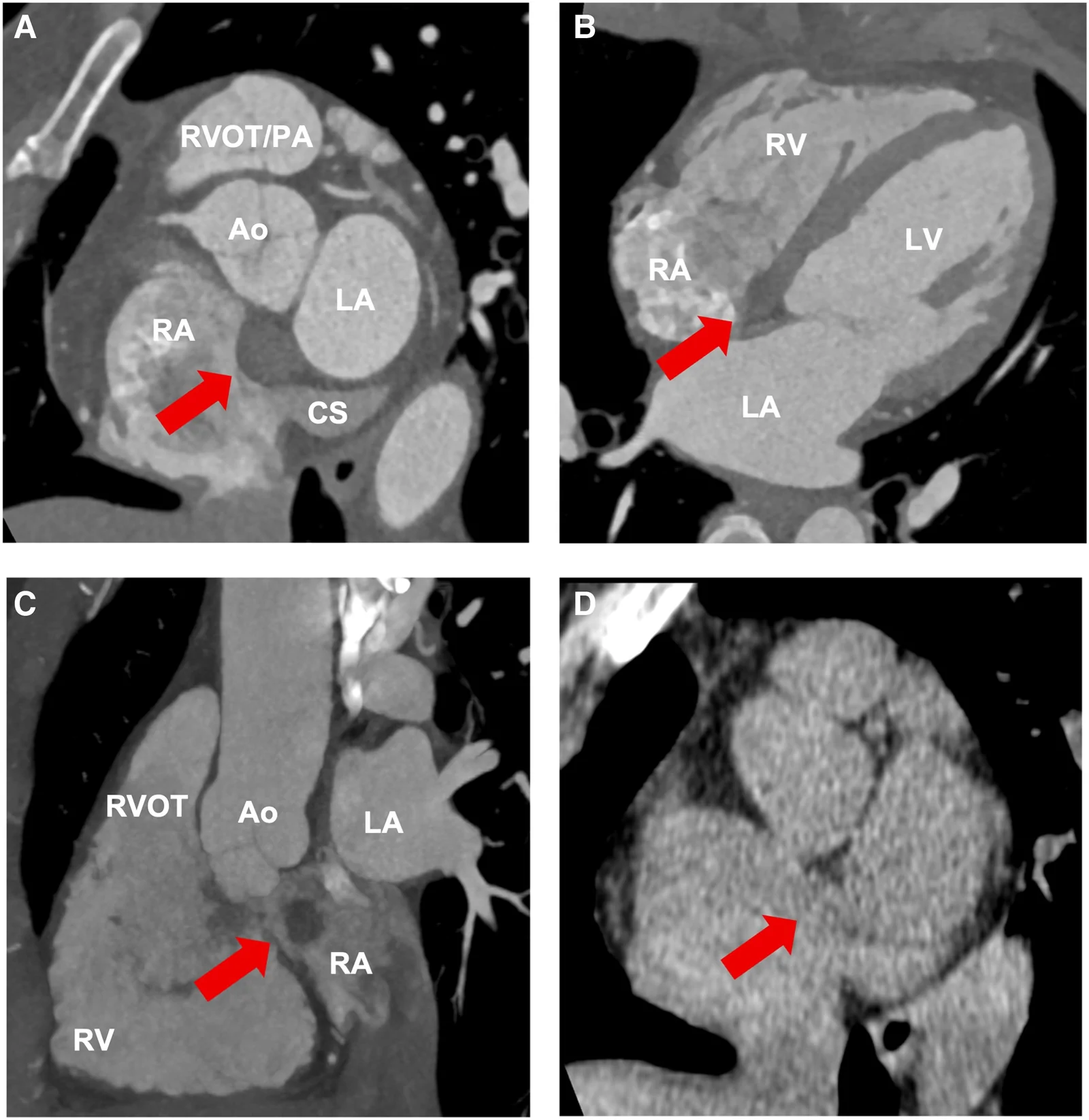
(*A–D*) Cardiac CT demonstrating a well circumscribed ovoid lesion in the right atrium.

Retrospective review of her initial transthoracic echocardiogram (*Figure 2A* and *B*) demonstrated the encapsulated right atrial lesion with a heterogeneous echolucent core. No flow turbulence was seen at the CS ostium. She had normal biventricular size and systolic function with satisfactory valvular function.

**Figures 2:**
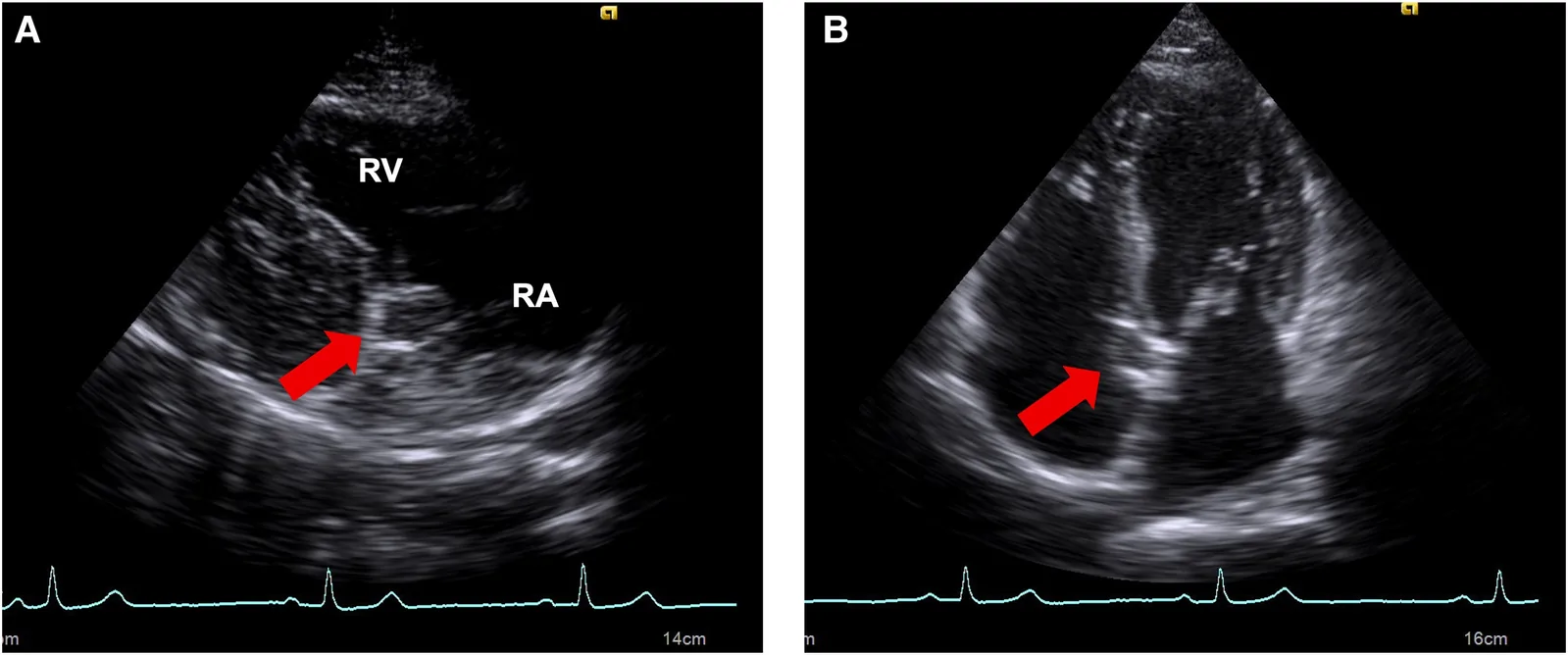
(*A* and *B*). TTE demonstrating a lesion with a heterogeneous echolucent core.

Catheter angiography demonstrated normal coronary arteries and no tumour blush. Cardiac magnetic resonance imaging (CMR) confirmed an encapsulated ovoid lesion which was hyperintense (to myocardium) on SSFP cine (*Figure 3A*), T1 (*Figure 3B*) and T2 weighted imaging. There was no lesion signal loss with SPAIR fat suppression (*Figure 3C*). On native T1 parametric mapping (3T Siemens MOLLI), low lesion times (mean 723 ± 10 msec vs. basal lateral LV 1160 ± 20 msec vs. anterior chest subcutaneous fat 300 ± 30 msec) were seen (*Figure 3D*). The lesion was imperceptible from blood pool on T2 mapping, precluding assessment of T2 times. Significant lesion vascularity was excluded with absence of enhancement on first pass perfusion (*Figure 3E*) and early gadolinium imaging. Late imaging showed scant punctate foci of heterogeneous enhancement (*Figure 3F*).

**Figure 3:**
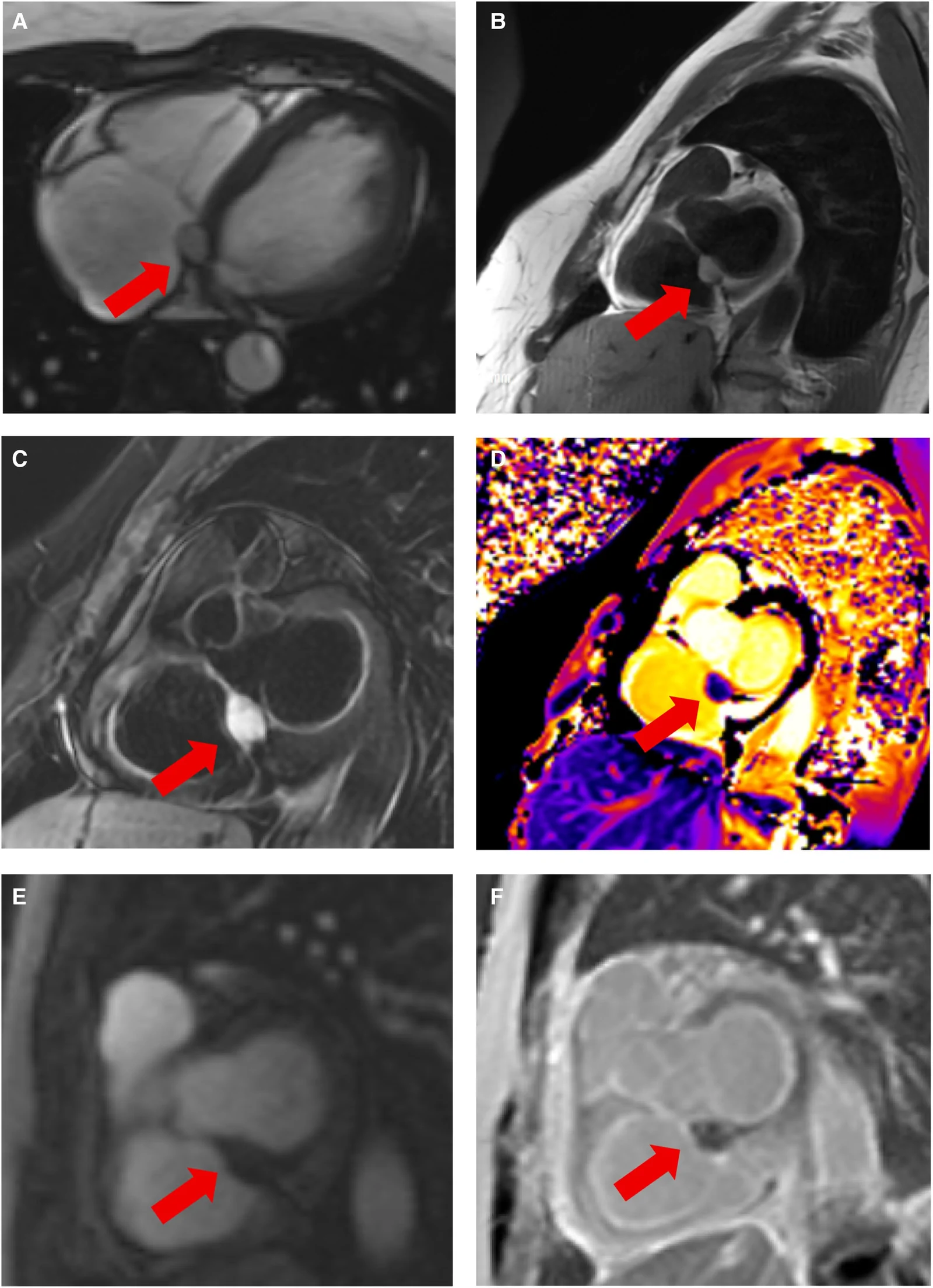
(*A–F*). Cardiac MRI demonstrating the right atrial lesion.

Given high risk of sudden cardiac death, she was referred for semi-urgent cardiac surgery. A median sternotomy was performed, and the right atrium was opened. A tumour, measuring 20 mm in maximal dimension, was noted in the AV node region located between the coronary sinus ostium and the tricuspid septal leaflet (*Figure 4*). The tumour was carefully removed from the surrounding tissue and grey murky fluid was extracted from the tumour. The interatrial septum was repaired with a bovine pericardial patch. Her post-operative course was complicated by complete heart block (CHB), requiring pacemaker implantation. Histology confirmed a collapsed cystic structure. The cyst lining was smooth. Microscopy showed fibrofatty tissue with cardiac muscle at the periphery, containing numerous cysts of variable size, lined by bland epithelium or reactive squamous epithelium. Patchy chronic inflammation was present, but there was no evidence of malignancy. The features were consistent with CTAVN (*Figure 5A–D*). Repeat transthoracic echocardiogram 1 year later was normal with no evidence of AV nodal tumour recurrence.

**Figure 4:**
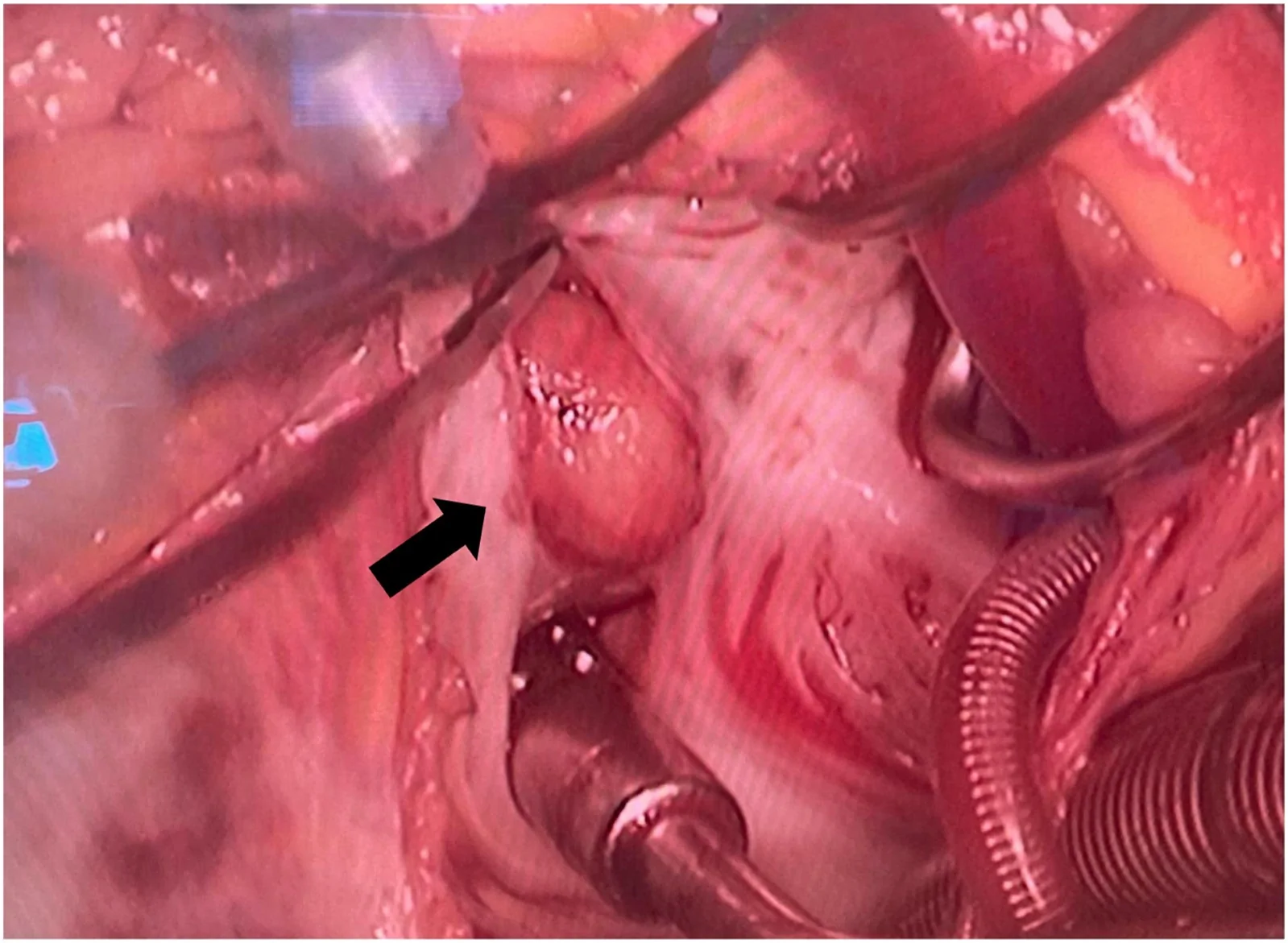
Surgery revealed a 20 mm tumour in the atrioventricular node region.

**Figures 5:**
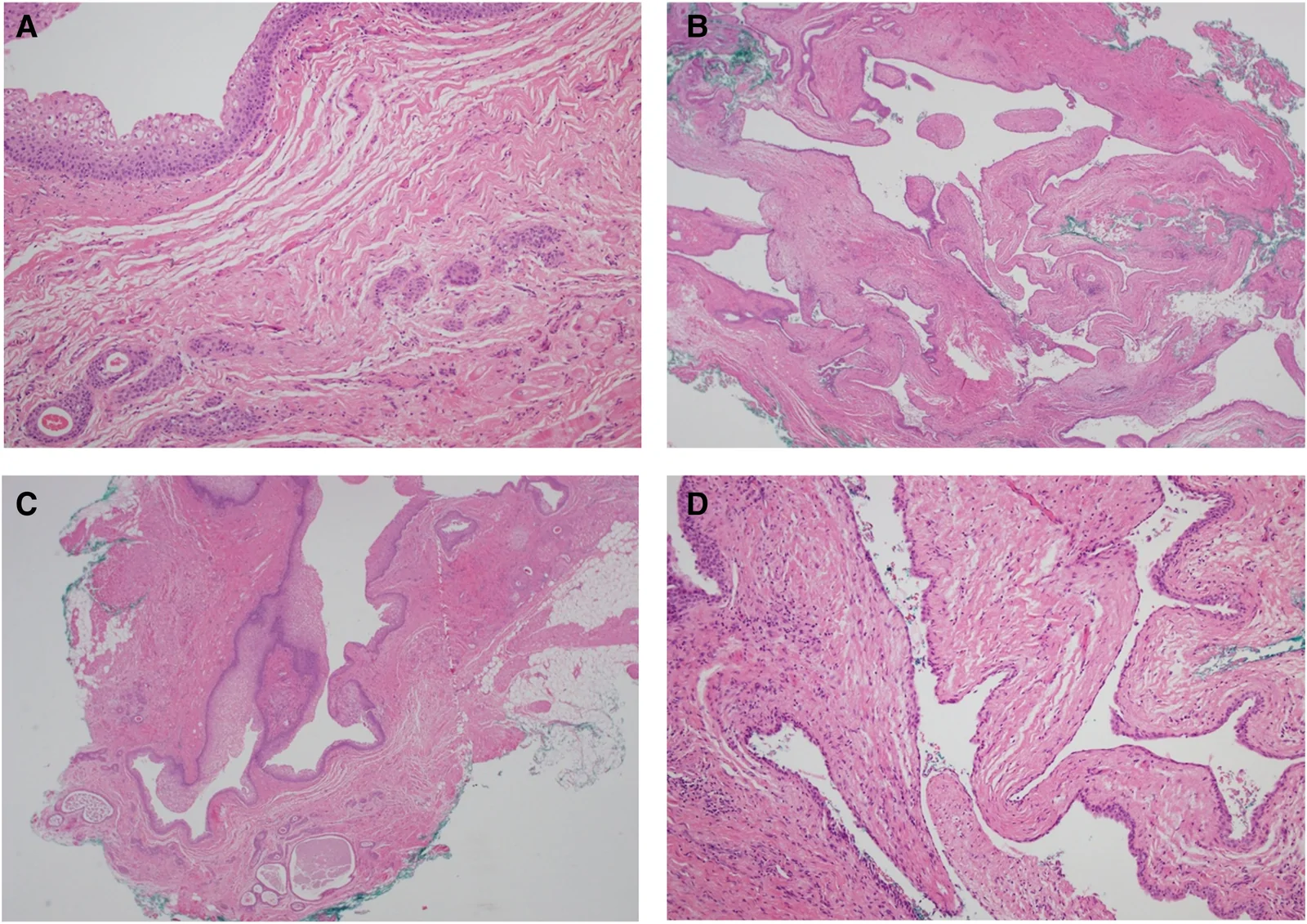
a-d. Histology confirmed cystic tumour of the atrioventricular node.

## Discussion

Cystic tumours of the AV node (CTAVN) are exceptionally uncommon congenital cardiac tumours, with an incidence of 0.0017%–0.03%, representing 2.7% of all cardiac tumours.^2^ The average age at diagnosis is 38 years, with a higher prevalence in females. However, these tumours are most commonly diagnosed during post-mortem examination.^1^ Very few reported CTAVN cases have been diagnosed antemortem and successfully treated with surgical resection.^3^ The tumours are typically located in the Triangle of Koch in the AV nodal region at the base of the atrial septum.^2^ Tumour size varies between 0.5 and 30 mm.^3^ Catheter angiography may demonstrate ‘tumour blush’ from the AV nodal artery or an altered course of the latter.

While the tumour is histologically benign, it confers high risk of sudden death due to CHB or ventricular arrhythmias.^1^ Given proximity to the AV node, CTAVN is associated with CHB in over 65% of patients and partial AV block in 15% of patients. It has also been reported in association with paroxysmal atrial arrhythmias, pre-excitation through left-sided accessory pathways and ventricular arrhythmias. In our case, CTAVN was associated with AVNRT. It remains unclear whether CTAVN has a direct arrhythmogenic effect or whether disruption of AV nodal conduction by the tumour unmasks underlying substrate for re-entrant arrhythmias^4^ Ventricular arrhythmias are believed to arise from impaired impulse conduction through abnormal AV junctional tissue, leading to ectopic foci that can precipitate ventricular tachycardia or fibrillation.^5^ An additional proposed mechanism is infiltration of tumour into the ventricular myocardium or Purkinje fibres.^1^ Common presentations include palpitations, chest pain, dyspnoea, dizziness, and syncope. Complications include myocardial infarction, stroke due to risk of atrial arrhythmias, and sudden cardiac death.

CTAVN can be seen on transthoracic or transoesophageal echocardiogram, but it is often difficult to visualize. Furthermore, it can be challenging to differentiate CTAVN from other tumours on echocardiography.^1^ CT and MRI play a central role in localizing and characterizing tumours. Intra-cardiac mass lesions have numerous differential diagnoses, including lipoma, myxoma, fibroma, and sarcoma.^6^ Most have distinct imaging features, for example, fat-signal suppression in lipomas and avid late enhancement in fibromas.^6,7^ With CMR morphologic assessment and tissue characterization, the ‘CMR mass score’^8^ for our case was 2 (1 point for sessile attachment and 1 point for heterogenous late enhancement) which favoured non-malignant histopathology.

The main differential for CTAVN is the comparably rare bronchogenic cyst (BC). Amongst intra-cardiac BCs, the interatrial septum is the most common site,^9,10^ accounting for 50%–75% of reported cases. Depending on intralesional protein content, CTAVN and BC may show overlapping T1 and T2 signal characteristics. Key discriminating considerations are:

1. BCs tend to be larger (several centimetres) than CTAVNs^10,11^
2. BCs lack vascularity^12,13^ and central enhancement, whereas CTAVNs characteristically shows some degree of enhancement—variable in intensity—on late gadolinium enhancement, owing to its solid epithelial component.^1,14^

Regarding CMR late enhancement specifically, we draw attention to the limited and heterogeneous evidence base and offer a histological rationale for the variable patterns reported. Our histologically confirmed case showed scant late enhancement, in contrast to the intense enhancement reported by Fiset *et al*.^14^ and Velasco *et al.*^1^ First, antemortem CTAVN cases characterized with contrast-enhanced CMR remain few, and enhancement has been described as a spectrum rather than a pathognomonic pattern; even in the well-characterized report by Fiset *et al.*, the lesion returned a heterogeneous T1 signal. Second, this variability is plausibly explained by differing proportions of solid epithelial nests, fluid-filled cysts, and fibrous stroma,^11^ such that the degree of enhancement reflects the ratio of vascular/solid tissue to non-enhancing fluid.

Parametric mapping may further aid tissue characterization. In our case, the lesion returned a native T1 of approximately 700ms—distinctly shorter than normal LV myocardium (1100 ms) and markedly shorter than the prolonged T1 expected of simple serous fluid, yet clearly longer than chest-wall fat (300 ms). This intermediate, shortened native T1 suggests complex proteinaceous content rather than the watery content of a simple serous BC, and additionally argues against pseudo-enhancement from a high intrinsic T1—thereby supporting our phase-sensitive inversion recovery finding of true, scant heterogeneous late enhancement.

Post-mortem studies indicate that device implantation does not avert sudden death risk in patients with CTAVN. Thus, surgical resection has been the management strategy in most antemortem cases reported to date.^2,15,16^ However, due to the rarity of CTAVN, evidence remains limited and there are currently no standardized guidelines that define optimal management, nor a standardized approach to surgical resection.^17^ While complete resection is generally preferred, partial excision has been reported in some cases to minimize potential injury to the conduction system. There are also reports describing a minimally invasive technique using an anterior minithoracotomy with peripheral cardiopulmonary bypass.^2,3^ AV node dysfunction requiring pacemaker implantation is common after surgery.

## Conclusions

CTAVN is associated with a high risk of sudden cardiac death. Thus, early detection, accurate diagnosis using cardiac CT and MRI, and timely surgical resection are essential. While rare, CTAVN should be considered in young adults who present with AV block.

## Data Availability

The data underlying this article are available in this article.

## Lead author biography

Jessica Yao is a cardiologist with a subspecialty interest in Cardiac Imaging and Cardio-obstetrics.

## Notes

**Consent:** The patient has given consent for publication of this case report. This case report complies with the COPE guidelines.
